# Student Performance During a Simulated Patient Encounter Has No Impact on Debriefer Adherence to PEARLS Debriefing Model

**DOI:** 10.1007/s40670-021-01290-2

**Published:** 2021-04-23

**Authors:** Richard McNutt, Matthew Tews, A. J. Kleinheksel

**Affiliations:** 1 Department of Emergency Medicine, Medical College of Georgia at Augusta University, 1120 15th Street, AF 1018, 30912 Augusta, Georgia; 2 Department of Medicine, Medical College of Georgia at Augusta University, Augusta, Georgia

**Keywords:** Simulation, Debriefing, PEARLS, Emergency medicine, Asthma exacerbation

## Abstract

**Purpose:**

Debriefing is necessary for effective simulation education. The PEARLS (Promoting Excellence and Reflective Learning in Simulations) is a scripted debriefing model that incorporates debriefing best practices. It was hypothesized that student simulation performance might impact facilitator adherence to the PEARLS debriefing model. There are no published findings on the effect of student performance on debriefer behavior.

**Methods:**

Third-year medical students participated in a video-recorded, formative simulation to treat a high-fidelity mannequin for an asthma exacerbation. A faculty debriefer trained in the PEARLS model evaluated student performance with a standardized rubric and conducted a recorded debriefing. Debriefing recordings were analyzed for debriefer adherence to the PEARLS model. Debriefers were assigned a debriefing score (DS) from 0 to 13; 13 was perfect adherence to the model. Definitive intervention (DI) for asthma exacerbation was defined as bronchodilator therapy. Critical actions were as follows: a focused history, heart/lung exam, giving oxygen, and giving a bronchodilator.

**Results:**

Mean DS for the debriefers of students who provided DI was 8.57; 9.14 for those students who did not (*P* = 0.25). Mean DS for debriefers of students who completed all critical actions was 8.68; 8.52 for those students who did not (*P* = 0.62). Analysis of elapsed time to DI showed no relationship between the time DI was provided and DS.

**Conclusions:**

Student performance had no impact on debriefer performance, suggesting the PEARLS model is an effective aid for debriefers, regardless of learner performance. These findings suggest student performance may not bias facilitators’ ability to conduct quality debriefings.

## Introduction

Medical simulation is an application of experiential learning [1, 2]. Reflective practices, such as debriefing, are essential to the success of the experiential learning cycle [3]. In contrast to feedback, debriefing is bidirectional, interactive, specific, and focuses on reflection-on-action [4–7].

Existing literature suggests debriefing is a necessary component for learning in medical simulation. One randomized control trial of simulated airway management found that only simulation *in combination with debriefing*, maintained the skills learned during the course [8]. A cardiology review course using simulation with debriefing showed an improvement in clinical skills when compared to a historical comparison group who did not receive debriefing [9]. A meta-analysis of debriefing showed a 25% improvement in performance with debriefing [10]. A systematic review of medical simulation showed that debriefing was the single most important component to effective learning [11].

Debriefing should focus on learner development as opposed to assessment. A focus on assessment may impede learner development by making the learner feel defensive and less open to change, while also biasing debriefer behaviors [12]. In fact, studies of the impression-perseverance effect show that even when an observer knows that good or bad performance is a false condition, this biases the observer’s perception of competence [13, 14]. This presents a conundrum for a would-be debriefer. In order to provide effective debriefing, the debriefer must assess how well the learner performed the task(s), so as to focus the debriefing on ways for the learner to improve. Yet, too much focus on assessment may impede the learner’s receptiveness, as well as bias the debriefer. It was hypothesized that good or poor learner performance may exacerbate this problem by influencing debriefer behavior. At the time of this study, there was no known literature specifically addressing the impact of learner performance on debriefer behavior. Thus, it was hypothesized that good *or* poor student performance might improve *or* worsen debriefing behavior, *or* vice versa (i.e., no directional relationship was hypothesized due to the exploratory nature of the study).

Since debriefing is a skill that must be taught [15], the answer to this question has implications at both the individual facilitator and programmatic level. If good and/or poor student performance worsens debriefing quality, this would negatively impact learning outcomes [8–10]. Though this begs the questions: what constitutes a “good” debriefing, how does an individual debriefer achieve this, and how can it be scaled across an organization? While there are faculty development programs available [16, 17], there is no national standard for faculty training in medical simulation or debriefing, despite ample literature supporting specific debriefing best practices [8–11, 18–32].

The Promoting Excellence and Reflective Learning in Simulations (PEARLS) debriefing model is a scripted approach to simulation debriefing which offers sample phrases for each stage of the debriefing to assist facilitators in incorporating debriefing best practices [18–22]. In a randomized trial of scripted vs non-scripted debriefing in Pediatric Advanced Life Support (PALS), scripting increased student knowledge acquisition and team leader performance [21]. PEARLS scripting improves model accessibility for those debriefers still developing their debriefing skills [18]. PEARLS circumvents some limitations of other debriefing models, including the prioritization of expediency and a requirement for high levels of experience for successful application [20, 23–25]. The PEARLS model presents a sequence of four stages: (1) reactions, (2) description, (3) analysis, and (4) summary [18]. These stages and their associated sample phrases assist debriefers in setting the stage for the debriefing, organizing the debriefing, and helping debriefers pose questions [18]. The PEARLS approach combines the debriefing strategies of learner self-assessment [20, 24, 26], facilitated reflection and understanding [27–29], directed performance feedback [30, 31], and focused teaching [28, 32]. PEARLS contains these blended strategies within a consolidated model [18]. This gives debriefers several clear and actionable strategies to employ as they facilitate learning. The PEARLS framework also assists the debriefer in choosing the best suited educational approach(es) for their learners during the analysis phase [18].

The Medical College of Georgia (MCG) Educational Simulation Program provides support to MCG faculty in the design, development, and implementation of high-quality formative and summative educational simulation activities. The program also runs an annual mid-clerkship simulation in which approximately 200 medical students participate in an individual, formative simulation activity. In order to facilitate the training and development of debriefing best practices for the facilitators, the PEARLS model was selected after a needs assessment of the Educational Simulation Program at the medical school and an analysis of available debriefing models. MCG faculty had very little or no debriefing experience with the PEARLS model prior to its adoption. To prepare for the event, faculty facilitators were required to participate in simulation and debriefing training. Facilitators were first provided with asynchronous training materials, which included video-recorded examples of the asthma exacerbation case and PEARLS debriefing. Following the completion of the asynchronous training, facilitators attended individual and small-group in-person training in the simulation center, where they could walk through the case and debriefing model with the director of the simulation program and get hands-on experience with the simulation technology.

Given the variable simulation and debriefing experiences of the facilitators, the PEARLS model was selected by the program for the goal of improving consistency in facilitator debriefing practices, thereby providing more consistent learner experiences. This study evaluated the effects of student performance on debriefer behavior during the mid-clerkship simulation activity. For this study, PEARLS best practices were conceptualized as a proxy for debriefing quality. This study was conducted in order to better understand the relationship between student simulation performance and debriefer behaviors, as this relationship will have direct implications for the continued development of faculty debriefers [8–10].

## Materials and Methods

### Simulation Scenario Overview

The simulation case was a formative, non-graded, individual activity that was designed as an opportunity for student learning and reflection. This study was considered expedited by the institutional review board of Augusta University. All students were collectively oriented to the activity, briefed on the scenario parameters, and given an orientation to the simulation room and its capabilities. Each student then rotated through the same case: a patient exposed to outdoor smoke that triggered an asthma exacerbation. The patient had a history of asthma, presented in obvious respiratory distress with diffuse wheezes on exam in both lungs, and vital signs showing tachypnea and hypoxia. The student was instructed to play the role of the physician, evaluating the patient, ordering diagnostic tests, managing the asthma exacerbation, and then calling the patient’s primary care physician to arrange a disposition, after which the case was ended.

### Simulation Room Overview

The simulations were conducted in one of five dedicated simulation rooms designed to simulate an emergency department room. The patient was a high-fidelity mannequin with voice capability under manual control by the scenario facilitator. All students used the same kind of mannequin. Real-time vital signs were on display at all times. Results of tests were given as handouts to the student by a trained actor who played the role of the nurse in the room. Equipment and supplies available to the student included intravenous (IV) catheters and fluids, nasal cannula and oxygen mask, nebulizer to mimic albuterol, medications for IV or oral administration (i.e., prednisone). Consultations were made via a phone located in the simulation room.

### Simulation Scenario Facilitator and Actor

The scenario was facilitated from a control room by a trained faculty member who acted as the patient and the consultant. The control room was divided by a one-way glass or was a room with a monitor that projected the simulation room. A computer controlled the mannequin’s vital signs and lung sounds regardless of location. A push to talk microphone allowed the facilitator to voice the patient. A phone allowed the facilitator to act as the family physician who calls for a case presentation via phone. The nurse actor performed the student’s orders and provided the student with test results in the simulation room. Both the facilitators and nurse actors were trained on the scenario. The facilitators were also trained in the PEARLS debriefing model and given a PEARLS handout to follow during the debriefing. Each facilitator and nurse actor managed a single simulation encounter with a single student. After the scenario ended, the nurse actor stayed in the room for the next encounter, while the facilitator and student went to another room for debriefing.

A total of 32 faculty facilitators participated. Of these facilitators, five were non-physicians. These five facilitators were Doctors of Philosophy, one each in pharmacology, anesthesia, physiology, biochemistry, anatomy. Of the 27 physicians, the breakdown by specialty was: seven internal medicine, six pediatric emergency medicine, five emergency medicine, two surgery, two pediatrics, two family medicine, and three OB/GYN. The facilitators were of varied simulation experience. We assessed experience level of the facilitators using the Dreyfus model of skill acquisition [33]. The breakdown by competency was: 11 novice, nine advanced beginner, eight competent, two proficient, two expert.

### Simulation Scenario Assessment

Although this was a formative experience without any formal evaluation, the facilitator completed a case evaluation form for each student as the scenario unfolded. Key components of the form were a start time, end time, and time at which the bronchodilator was first given. The specific medication(s) and dose(s) were also recorded. The form was also used to record the order in which each action was taken in order to determine how students prioritized their decision-making (e.g., if the student’s first action was to gather an HPI, that would be marked “1,” and if they then ordered a nebulizer treatment that would be marked “2”). If an action was not performed, it was not assigned a number.

### Debriefing Assessment

A 15-min debriefing followed each 15-min simulation scenario. We chose a 1:1 time ratio for simulation to debriefing based on the importance of debriefing in the experiential learning cycle and the time allotted for the event [3, 8–11]. Both the scenario and debriefing were recorded. After developing a new, structured rubric to assess facilitator adherence to the PEARLS model, evidence of construct and content validity for the instrument was collected through a pilot test and expert review. Recordings of a prior simulation case debriefing were scored with the instrument as a pilot test, which also allowed for an estimate of reliability. Problematic items in the pilot test were revised for clarity. The revised instrument was then evaluated by experts using cognitive interviewing. Inter-rater reliability was established between all 32 facilitators with a correlation coefficient of 0.913. A primary researcher then observed each video and assessed how many PEARLS practices were performed during each debriefing. The instrument to assess adherence to the PEARLS model contained 13 observable facilitator behaviors. The debriefing scored one point for when the debriefer initiated the behavior and one-half point if the student initiated the behavior instead of the debriefer. A score of zero was assigned for no observable behavior. Thus, this analysis generated a debriefing score (DS) from 0 to 13 for each individual debriefing. Debriefing scores were then compared to the student evaluation forms in order to generate the results. The researcher that analyzed all of the videos was a medical student trained in the PEARLS debriefing model and use of the new PEARLS assessment rubric. The student was supervised by the last author as part of a student research program funded by the Medical College of Georgia.

## Results

### Debriefing Score

As noted above, each debriefer was assigned a DS for each debriefing they facilitated. Given that this score consisted of observed PEARLS best practices, the debriefing score was considered a proxy for both the debriefing quality and as an assessment of the debriefer’s behaviors; a higher score indicated both a higher quality debriefing and a greater adherence to the PEARLS debriefing model. This was based on the idea that each PEARLS component contributes to a better debriefing, and therefore, improves learning [18–32]. Table 1, PEARLS debriefing items, lists each item in the PEARLS debriefing model along with the respective number of times Facilitators Initiated (FI) and Students Initiated (SI) each debriefing item (out of a total of 187 debriefings). Table 1 also has a combined (C) column. This column adds together the FI and SI instances of each item, to give a total number of times each item was performed. Debriefers performed each item the majority of the time with the exception of item 4 (“Asked the student to summarize the case”), item 10 (“Used preview statements to introduce new topics”), and item 13 (“Clearly ended the debriefing”).

**Table 1:** PEARLS debriefing rubric and items performed. Listed are each PEARLS debriefing item and the number of times each item was performed in total (combined), as well as whether or not the item was initiated by the facilitator or the student. The data was derived from the simulation debriefing of 187 3^rd^ year medical students at Augusta University in 2019

PEARLS item #	Description	Facilitator Initiated (FI)	Student Initiated (SI)	Combined (C)
1	Stated purpose and goals of the debriefing (e.g., “The purpose of this debriefing is to ensure that you get the most value possible from your simulation experience.”)	152 (81%)	0 (0%)	152 (81%)
2	Assured student of confidentiality (e.g., “Everything you say is off the record.”)	148 (79%)	1 (1%)	149 (80%)
3	Asked the student about their emotions or initial reaction (e.g., “How do you feel?” or “How do you think it went?”)	181 (97%)	1 (1%)	182 (97%)
4	Asked the student to summarize the case (e.g., “Tell me about what happened.”)	6 (3%)	3 (2%)	9 (5%)
5	Asked the student to identify strengths in their performance (e.g., “What went well?”)	109 (58%)	7 (4%)	116 (62%)
6	Described positive aspects of the student’s performance (e.g., “I noticed that you did ___ well.”)	167 (89%)	9 (5%)	176 (94%)
7	Asked the student to identify areas for improvement in their performance (e.g., “What would you do differently?”)	132 (71%)	22 (12%)	154 (82%)
8	Provided directive feedback or redirection for behaviors that were incorrect or suboptimal that were not identified by the student (e.g., “Next time you might want to….”)	176 (94%)	2 (1%)	178 (95%)
9	Asked student for thoughts or rationale during the case (e.g., “What were your thoughts when that happened?”)	111 (59%)	7 (4%)	118 (63%)
10	Used preview statements to introduce new topics (e.g., “At this point I’d like to take some time to talk about…”)	34 (18%)	0 (0%)	34 (18%)
11	Provided the student with an opportunity to reflect on their take aways/lessons learned (e.g., “So what did you learn today?” or “It sounds like next time you will…”)	124 (66%)	1 (1%)	125 (67%)
12	Asked the student if they had any questions or other topics they would like to discuss (e.g., “Do you have any questions before we end?”)	147 (79%)	2 (1%)	149 (80%)
13	Clearly ended the debriefing (e.g., “That concludes the debriefing.” or “Thank you for participating, I hope you found this useful.” or “We’re done.”)	92 (49%)	0 (0%)	92 (49%)

### Definitive Intervention

For the purpose of this analysis, bronchodilator administration was defined as the definitive intervention (DI) for the scenario [34]. The DS for the debriefer for those students who gave a bronchodilator during the scenario was compared to the DS for the debriefer of those students who failed to give a bronchodilator. Table 2, comparison of learner performance to debriefing score, shows mean DS for the debriefer of those students who gave a bronchodilator compared to those that did not. The mean DS for the debriefer of those students who gave a bronchodilator was 8.57 (2.22) with *n* = 180 (96%). The mean DS for the debriefer of those students who did not give a bronchodilator was 9.14 (2.17) with *n* = 7 (4%). The *p* value between means was 0.25.

**Table 2:** Comparison of learner performance to debriefing score. This table details the number of students who gave, and failed to give, bronchodilator treatment. It compares the mean debriefing score between these two groups. It also details the number of students who completed all critical actions and those who failed at least one critical action. It compares the mean debriefing score between these two groups. The data was derived from the simulation performance and subsequent debriefing of 187 3rd year medical students at Augusta University in 2019

Number of learners that gave definitive intervention	180 (96%)
Mean debriefing score when definitive intervention given	8.57 (2.22)
Number of learners that did not give definitive intervention	7 (4%)
Mean debriefing score when definitive intervention not given	9.14 (2.17)
Number of learners that completed all critical actions	88 (47%)
Mean debriefing score when all critical actions completed	8.68 (2.16)
Number of learners that failed one or more critical actions	99 (53%)
Mean debriefing score when failed one or more critical actions	8.52 (2.28)

### Critical Actions

For the purpose of this analysis, critical actions were defined as follows: (a) conduct HPI, (b) conduct heart exam, (c) conduct lung exam, (d) order bronchodilator treatment, or (e) order O2 [34]. The critical actions were not defined prior to the simulation exercise and were not communicated as such to students during debriefing. Making a disposition for the patient was not considered a critical action because a disposition discussion with the patient’s primary physician was scripted into the scenario in order to provide every student an opportunity to present the patient as part of the simulation. Table 2 shows mean facilitator DS for debriefers of those students who completed all five critical actions versus those students who failed to complete all critical actions. The mean DS for debriefers of those students who completed all critical actions was 8.68 (2.16) with *n* = 88 (47%). The mean DS for debriefers of those students who did not complete all critical actions was 8.52 (2.28) with *n* = 99 (53%). The *p* value between means was 0.62.

### Time to Definitive Intervention

**Fig. 1:**
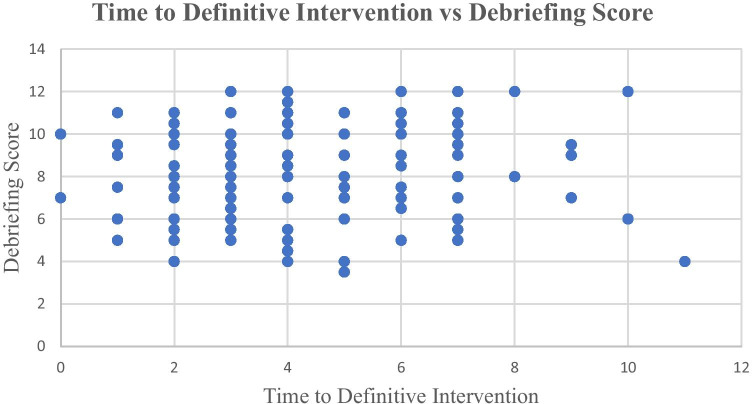
Time to definitive intervention vs debriefing score. This graph compares the time when the learner gave a bronchodilator, to the debriefing score assigned to the learner. The time intervals were recorded by debriefers at 1-min intervals. A time score of 0 indicates the student administered a bronchodilator at a time less than 1 min after case start. The data was derived from the simulation performance and subsequent debriefing of 187 3rd year medical students at Augusta University in 2019

**Fig. 2:**
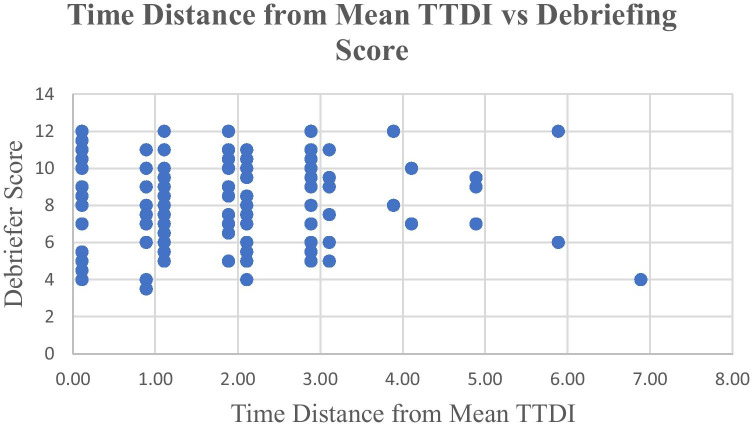
Time distance from mean TTDI vs debriefing score. This graph compares debriefing score to the time difference (sooner or later) between the time at which the learner gave a bronchodilator and the mean time of bronchodilator administration. The mean time was calculated using only the 180 learners who gave a bronchodilator. The data was derived from the simulation performance and subsequent debriefing of 187 3rd year medical students at Augusta University in 2019

The time at which the student gave a bronchodilator was defined as the Time to Definitive Intervention (TTDI). A desired TTDI was not determined prior to the simulation and was not communicated to students during debriefing. The time intervals were recorded by debriefers at 1-min intervals. A time score of 0 indicates the student administered a bronchodilator at a time less than one minute after case start. Plotting TTDI and DS produces the scatter plot shown in Fig. 1—TTDI vs DS. There was no discernable relationship identified between the two variables. DS was further analyzed as a function of the time difference between the student’s TTDI and the mean. The mean TTDI was 4.11. The hypothesis was that it might not be absolute performance that influenced debriefer behavior, but deviation from mean performance that influenced debriefer behavior. When the time distance between mean TTDI was plotted against DS, it produced Fig. 2—time distance from mean TTDI vs DS. As with TTDI vs DS, this analysis showed no discernable relationship between the metric and DS.

## Discussion

This study was conducted in order to better understand the relationship between student simulation performance and debriefer behaviors. To assess debriefer behaviors, a new, reliable instrument was created to measure debriefer adherence to the PEARLS debriefing model. Analysis was conducted on the number of PEARLS observable best practices that occurred during the debriefing, for which a DS was assigned. The DS was then compared to measures of student performance, including DI by the student, which was defined as giving a bronchodilator medication; how quickly the student provided DI, termed TTDI; and if the student completed all critical actions (i.e., conduct HPI, conduct heart exam, conduct lung exam, give oxygen, give DI). Overall, the data showed that student performance has no effect on DS, and by proxy, no impact on debriefing quality or debriefer adherence to the PEARLS model.

Bronchodilator administration was used as the DI because bronchodilator administration is the sole intervention that could reverse or ameliorate the simulated patient’s underlying pathology of an asthma exacerbation [34]. Given how crucial bronchodilator administration is for an asthma exacerbation, it was hypothesized that students failing to provide a bronchodilator would influence DS. Of all the available performance metrics, this provided the starkest contrast between ideal and suboptimal performance. Suboptimal student performance might concern the debriefer, leading them to deviate from the PEARLS model and engage in more didactic teaching behaviors. However, poor student performance could have also motivated the debriefer to rely more heavily on the sample phrases. No directional relationship was hypothesized due to the exploratory nature of the study. However, despite the initial hypothesis, no relationship was identified between DS and providing, or failing to provide, DI.

While bronchodilator administration was defined as the DI, four other critical actions were identified as necessary for appropriate diagnosis and treatment of an asthma exacerbation [34]. The data was split approximately evenly between those students who performed all critical actions (*n *= 88, 47%) and those who did not (*n* = 99, 53%). Comparison of these cohorts showed even less evidence of a difference in DS and also reversed the trend seen in the DI comparison, with a slightly higher mean DS for debriefers of the students who completed all critical actions.

It was also hypothesized that TTDI might influence debriefer model adherence. A small TTDI was conceptualized as a better performance, as a debriefer might feel concerned when having to wait for a student to figure out what to do (i.e., provide DI) and this reaction might influence debriefer performance. As with the analysis above, it was hypothesized that a delay in TTDI might improve or degrade debriefer adherence. However, comparison of TTDI to DS indicated no relationship. It was considered that it might not be better student performance, per se, that influenced the debriefer adherence to the PEARLS model; it might be deviation from average performance (i.e., either exceptionally high performance or particularly poor performance) that influenced debriefer behavior. This prompted analysis comparing time distance from mean TTDI to DS. However, once again, this comparison showed no relationship.

This study demonstrates that student performance does not influence debriefer performance when the debriefer is using the PEARLS debriefing model. These results can be generalized to other simulation programs. The student body at the Medical College of Georgia is diverse in race, gender, nationality, and socioeconomic status. The faculty debriefers were similarly diverse. They included members of several medical specialties, included practicing clinicians, and basic science researchers. Debriefers also had a broad range of simulation and debriefing experience. See Methods for a detailed breakdown.

Facilitators performed the majority of the PEARLS items the majority of the time. However, facilitators performed items 4, 10 and 13 (Table 1) at the lowest frequency. The action performed least frequently was item 4, “Ask the student to summarize the case.” The reason for this is unclear. The preceding item asked the student to discuss how they felt about the case. Depending on how the student responded to this question, the facilitator may have perceived the student as summarizing the case in their response to this request (item 3 was performed in 182 of 187 debriefings, 97%). Also, items 5–7 refer to student’s areas of strengths and areas for improvement. The student’s discussion of their feelings about the case, may have led naturally to a discussion of strengths and weaknesses, thereby causing the debriefing conversation to naturally flow past the opportunity for a case summary. Similarly, item 10, “Used preview statements to introduce new topics,” was rarely performed (34 of 187 debriefings, 18%). Again, the reason is unclear. It may be that preview statements are more formal than is typical of normal conversational flow. It may be that natural transitions often occurred during debriefings, seeming to obviate the need for preview statements, or facilitators were not practiced at utilizing this technique for changing subjects. Facilitators performed item 13, “Clearly ended the debriefing,” a little less than half the time (92 out of 187, 49%). The reason for this is unclear. It may be that the facilitator was rushed by the end the debriefing, and/or felt the debriefing had clearly ended based on non-verbal cues.

A limitation of this study is the lack of a control group. Future research could include data collection on the behaviors of debriefers trained in and using a different debriefing model, trained debriefers using no identified debriefing model, and untrained debriefers without an identified model. However, this study does indicate that when the PEARLS debriefing model is used, students at all performance levels received debriefings of similar quality. Because prior research showed that debriefing models improve student learning [8–11, 18–32], and that debriefing training is necessary to achieve these benefits [15–17], it is reasonable to conclude that using the PEARLS model may help debriefers provide quality debriefings to students at all performance levels. Another limitation in the data is that only seven of the students failed to provide DI. There was a non-significant trend towards improved DS for those students who failed to provide DI. A larger data set may identify a difference in DS for debriefings of students with suboptimal performance. However, the analysis of critical actions suggests this is unlikely, given that when there are similar n values between cohorts, the difference between cohorts narrowed and the trend towards improved DS for worse performance was reversed. A further limitation in this study is it analyzes only specific, objective performance measures. The measurement included binary actions recorded or not observed, the order of actions performed, and time when each action occurred. What this study did not include was a measurement of students’ interpersonal interactions with the patient, nurse, and consultant. These behaviors could affect debriefers overall gestalt of “good” vs “poor” performance. For example, a student could have a low TTDI and complete all critical actions while being brusque and uncaring towards the patient and rude towards the nursing staff and consultant. This would likely be perceived by the debriefer as “poor” performance despite “good” metrics. Another confounder is the student’s reaction to the debriefing, as this could significantly impact debriefer performance. Future studies should assess debriefer perceptions of the student after conducting the debriefing to assess whether students’ interactions with the patient and staff were appropriate, and/or, student reactions and openness to the debriefing.

## Conclusions

In conclusion, this study demonstrated that student performance does not affect debriefer adherence to the PEARLS debriefing model. The analysis examined student performance through a conceptualization of ideal performance as definitive intervention and completion of critical actions. This study also explored any relationship between debriefer adherence and the time it took students to provide definitive intervention. This suggests use of the PEARLS model may help debriefers provide quality debriefing regardless of student performance.
